# Protein subfamily assignment using the Conserved Domain Database

**DOI:** 10.1186/1756-0500-1-114

**Published:** 2008-11-14

**Authors:** Jessica H Fong, Aron Marchler-Bauer

**Affiliations:** 1 National Center for Biotechnology Information, National Library of Medicine, National Institutes of Health, 8600 Rockville Pike, Bethesda, MD 20894, USA

## Abstract

**Background:**

Domains, evolutionarily conserved units of proteins, are widely used to classify protein sequences and infer protein function. Often, two or more overlapping domain models match a region of a protein sequence. Therefore, procedures are required to choose appropriate domain annotations for the protein. Here, we propose a method for assigning NCBI-curated domains from the Curated Domain Database (CDD) that takes into account the organization of the domains into hierarchies of homologous domain models.

**Findings:**

Our analysis of alignment scores from NCBI-curated domain assignments suggests that identifying the correct model among closely related models is more difficult than choosing between non-overlapping domain models. We find that simple heuristics based on sorting scores and domain-specific thresholds are effective at reducing classification error. In fact, in our test set, the heuristics result in almost 90% of current misclassifications due to missing domain subfamilies being replaced by more generic domain assignments, thereby eliminating a significant amount of error within the database.

**Conclusion:**

Our proposed domain subfamily assignment rule has been incorporated into the CD-Search software for assigning CDD domains to query protein sequences and has significantly improved pre-calculated domain annotations on protein sequences in NCBI's Entrez resource.

## Background

**Figure 1:**
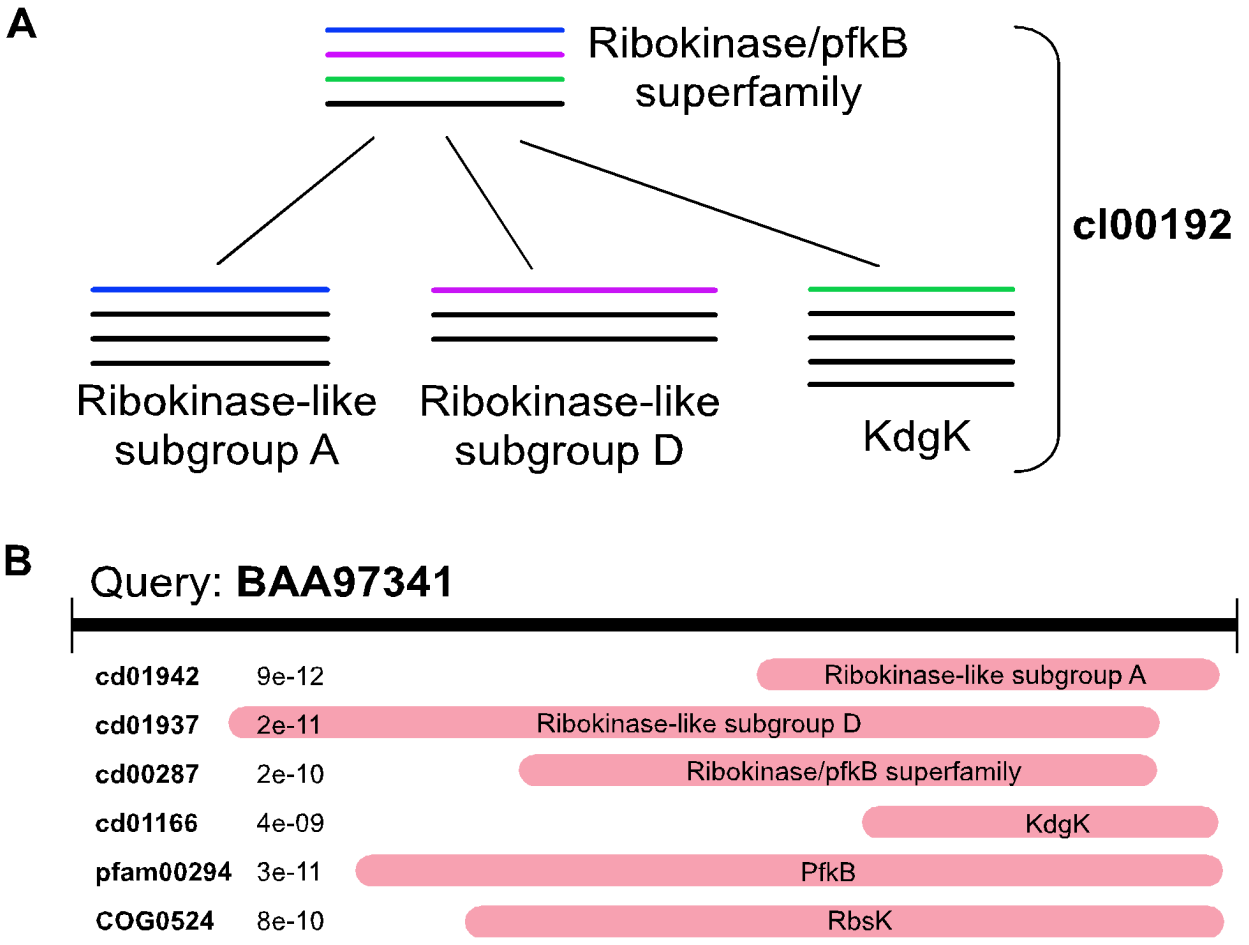
**Domain assignment among closely related domains**. (A) Schematic of NCBI-curated hierarchy illustrating domain models from the Ribokinase/pfkB superfamily (cl00192). Each domain model is pictured as a multiple sequence alignment. Parent and child models are defined to share at least one (overlapping) sequence (blue, purple, and green lines). The root domain of this hierarchy represents the whole superfamily and provides information about the conserved core regions and sequence variation within the superfamily. Its many subfamilies include the ribokinase-like subgroups A and D and KdgK. Ideally, a query sequence is labelled by the most specific domain that matches the sequence and that domain would yield the most significant hit. (B) A partial list of domain hits to query protein [Entrez:BAA97341] with domain accessions, names, and the E-values of their RPS-BLAST alignments to the query sequence. Previously, BAA97341 would have been assigned the domain with lowest E-value, cd01942. Our proposed method assesses the hit to cd01942 against a pre-computed, domain-specific threshold to determine that the hit with lowest E-value is not significant enough to be a confident match. Instead, we label the sequence generically by the superfamily of the best-matching domain, or cl00192.

A major goal in the post-genomic world is to infer protein function from sequence information. One popular approach is to classify protein families or domains by grouping homologous sequences and annotating the groups with properties such as general function, intracellular location, three-dimensional structure, conserved sequence patterns or motifs, evolutionary origin, and binding and active sites. Novel proteins can be characterized quickly by assigning a group via profile search methods. However, more than one family or subfamily may exhibit similarity to overlapping sequence intervals and to a degree that seems convincing (Figure 1). Assigning the protein to the correct group not only yields the correct annotations, but may also help to avoid propagating annotation errors and alleviate current issues with mislabelling in protein sequence databases [1,2]. Here, we examine the problem of making correct domain assignments from the Conserved Domain Database [3,4].

Domains are evolutionarily conserved units in proteins and frequently correspond to recurrent structural and functional units. The particular function of a protein depends on its combination of domains; two-thirds of prokaryotic proteins and 80% of eukaryotic proteins have more than one domain. To create new protein functions, novel domain architectures arise through domain rearrangement and recombination, frequently through gene duplication and fission or fusion events [5,6]. A domain may be represented as a multiple sequence alignment (MSA) of homologous sequence fragments. To identify the domains in a query protein sequence, the MSAs are converted into scoring models such as hidden Markov model or position-specific scoring matrix for use with database search algorithms such HMMER [7] and RPS-BLAST [8].

To refine protein annotation, domains models may be subdivided to represent more specific functions or conserved features. CDD curators apply phylogenetic and structural analysis to construct hierarchies of homologous domain models, related by common descent, to reflect aspects of their evolutionary histories [3,4]. Curation follows an iterative procedure to split domain models into subfamilies that redistributes sequences into more narrowly defined models. In the hierarchy tree structure, the leaf domains represent highly conserved and often orthologous protein subgroups. Their precursor (internal) domains, on the other hand, reflect ancient gene duplication events, as CDD aims to categorize ancient conserved domain families.

It may seem natural that once the profiles have been defined, the most significant match to a query sequence is the correct one. Indeed, domains from Pfam [9,10] and SMART [11] are assigned following the lowest alignment E-value that exceeds a family-specific cutoff [12,13]. This straightforward approach works well when the candidate domains are disjoint. Domain subfamilies may be obtained through automated methods such as the SCI-PHY algorithm for identifying functional subtypes of known domain families [14,15] or by mirroring other hierarchical domain classifications such as SCOP [16] and CATH [17]. However, subfamily assignment methods generally attempt to classify a member of a family at the subfamily level given that the family is known, as in a statistical pairwise/profile method proposed for SUPERFAMILY [18-20].

The systematic arrangement of CDD domains requires identifying the most suitable level of resolution among domain models that offer more or less fine-grained descriptions of a protein. We take the viewpoint that if a protein cannot be associated unambiguously with a specific subgroup or may be a member of a subgroup that has not been defined, the protein can be assigned a more generic domain model or the superfamily in general. Consequently the ideal domain assignment to a query sequence will be the most specific domain, within a candidate hierarchy, with a strong match to the sequence. Here, we analyze a set of correct domain assignments from CDD to establish an improved method for assigning domains to query sequences. The effectiveness of a traditional alignment score and domain-specific threshold is of particular interest, as this method is efficient and makes use of alignment information that is already computed for CDD.

### Constructing a benchmark set of correct domain assignments

To benchmark domain assignment heuristics, a reference set of domain assignments is constructed from the NCBI-curated portion of CDD v. 2.12. This set contains every sequence fragment present among the MSAs and its domain assignment. The NCBI-curated domains have undergone rigorous testing to optimize the MSAs and distributions of representative sequence fragments.

The correct or most specific domain for each sequence in a hierarchy is defined as the domain having no descendant that contains an overlapping sequence interval. Two sequence intervals from one protein are said to overlap if one sequence interval contains at least 30% of the positions of the other. While each sequence has been placed in the most specific domain model that characterizes it, this step is required as parent and child domains share overlapping sequences (Figure 1). Sequences with overlapping regions from more than one hierarchy are counted once for each hierarchy. Non-overlapping regions of a protein are treated independently. Alignments between all NCBI-curated domains and proteins present in the public Entrez protein set at time of analysis (September 2007) [21] have been pre-computed using RPS-BLAST. In this analysis, the alignment score refers to the bitscore, a normalized version of the raw alignment score between the query sequence and the PSSM, which allows alignments from different searches to be compared. The bitscore corresponds roughly to the alignment E-value and is used instead to avoid real value rounding issues.

A significant PSSM-sequence alignment is called a *hit*, for brevity. We call a match between a sequence region and its correct domain a *self hit *to distinguish it from other hits to overlapping sequence regions. Other hits to the sequences in the reference dataset serve as examples of incorrect domain assignments.

CDD v 2.12 contains 3078 NCBI-curated domains in 495 hierarchies, including 298 single-domain "hierarchies" and 197 trees with 2357 leaf and 423 internal domains. Many sequence fragments used to construct the NCBI-curated domain profiles come from proteins that have been replaced with newer versions or declared obsolete. Among the 109186 representative sequences in NCBI-curated domain hierarchies, over 21% have no hits and more than 90% of those sequences are no longer present in Entrez. This analysis excludes the 149 curated domains without corresponding live data in Entrez, leaving 2929 domains.

### Performance of a simple high-score assignment method

We begin by assessing the performance of the previous method for assigning NCBI-curated domains from CDD. The NCBI CD-Search tool [22] has historically relied on alignment E-value and properties such as the source domain database to highlight one or a few most likely domain assignments, without claiming to pinpoint the correct domain assignment. Analysis of all hits to the sequences in the benchmark set reveals that assigning domains by high alignment score alone achieves 96% accuracy over all sequences and 100% accuracy over the representative sequences for 91.5% of domain models. Further, categorizing non-self hits by their hierarchical relationships to the correct domain reveals that assigning to a subclass of the correct domain is the most common type of error when a sequence matches the correct domain and other domains (Table 1). For simplicity, all non-self hits are labelled as incorrect hits in the tables although some child/descendant and parent/ancestor assignments may not be regarded as actual classification errors. Child/descendant domains score higher than the self hit for 21.8% of sequences with both types of hits. These higher scores may reflect computational bias from longer profiles, overly cautious assignment of a sequence to a more generic domain, or missing subfamilies. In contrast, higher scores from parent/ancestor domains or domains from other branches of a hierarchy are rarely observed. For additional data and discussion of all analyses described in this document, see [Additional file 1].

**Table 1:** Rate of incorrect domain assignment by type of other domain

(A) Type of incorrect hit	(B) # Sequences with self hit and other hit (# domains)	(C) # Sequences with higher score from other hit (# domains)	(D) Error rate
Self only	23918 (264)	-	-
Parent/ancestor	49934 (2402)	137 (58)	0.35%
Child/descendant	9822 (274)	2135 (129)	21.8%
Other domain in hierarchy	47362 (2306)	100 (37)	0.15%
Domain outside hierarchy; sequence not in other domain model	6747 (506)	421 (32)	3.0%
Domain outside hierarchy; sequence in other domain model	736 (66)	313 (18)	21.1%

(A) Classes of incorrect hits, labelled by hierarchical relationship to the specific domain. (B) Number of sequences with self hit and other hit of the respective category; the number of domains containing these sequences is in parentheses. (C) Number of sequences with other domain scoring higher than correct domain; the number of domains containing these sequences is in parentheses. (D) Error rate as percent of sequences with higher score from incorrect domain than the self hit, averaged over domains containing sequences with both types of hits.

We define a score threshold for each domain to be the lowest self-hit score to that domain among all of its sequences in the benchmark set. This additional heuristic, in particular, reduces incorrect assignments to subclasses as only 9.1% of hits to subclasses score above the thresholds for those subclasses (Table 2). The threshold definition works around the issue of small data size–over 60% of domains have 20 or fewer self hits–and addresses variances in scores between domains due to properties such as length and residue composition, or practical issues such as incomplete local alignments, which are not considered by simple high-score heuristics. The definition is more restrictive than its Pfam counterpart, the minimum alignment score among all sequences in the automated "full alignment", as NCBI-curated hierarchies in CDD tend to present a finer-grained classification of a protein domain family.

**Table 2:** Incorrect domain hits with alignment scores above domain-specific thresholds

(A) Type of incorrect hit	(B) Domains with incorrect hits	(C) No incorrect hits above threshold	(D) With incorrect hits above threshold	(E) Incorrect hits above correct domain threshold	(F) Incorrect hits above hit domain threshold
None	9.0%	264	-	-	-
All incorrect hits	91.0%	1778	887	9.1%	25.9%
Parent/ancestor	82.0%	1838	564	9.0%	82.3%
Child/descendant	9.4%	59	215	42.8%	9.1%
Other domain in hierarchy	78.7%	1939	367	2.7%	5.5%
Domain outside hierarchy	17.3%	444	64	9.3%	29.7%

(A) Classes of incorrect hits, labelled by hierarchical relationship to the specific domain. (B) Percent of domains with each type of incorrect hit to its representative sequences, among a total of 2929 domains. (C) The number of domains without incorrect hits that score above the correct domain's threshold and (D) with incorrect hits that score above the correct domain's threshold. (E) Percent of non-self hits that score above the correct domain's threshold or (F) the hit domain's threshold. In (E-F), the percent is averaged over domains with the respective class of non-self hits.

### Proposed rule for specific domain assignment

We propose to label a single domain as correct or specific for a protein sequence region if its alignment score is highest among all domains that align to overlapping regions of the protein sequence and the score exceeds a pre-calculated threshold for the domain, defined as the minimum alignment score among confirmed members of the domain. Sequence intervals that are difficult to group with a specific subclass with high confidence following this rule may receive only generic domain assignments. Assuming that the set of overlapping domains represents an ancient domain superfamily, such a generic assignment would be characterized as membership with the respective superfamily.

### Reducing misclassifications and errors due to missing subfamilies

A more concrete picture of the effect of the proposed rule may be gleaned by quantifying misclassifications, defined to be either descendants of the correct domain or domains that lie in other branches of the correct hierarchy. Averaged over domains in multi-domain hierarchies and counting only sequences with self hits, the misclassification rate using high scores only is 2.6%. Incorporating score thresholds to eliminate low-scoring best hits reduces the misclassification rate to 0.85%. Misclassifications may also be used to estimate error due to missing subfamilies. Not all subclasses in a domain hierarchy may have been identified as the available sequence databases only provide a terse snapshot of protein domain diversity. We simulate a cross-validation experiment to ask, if an existing domain model were missing from a hierarchy, what fraction of its sequence intervals have best hits to other models in the hierarchy that are not ancestors of the correct model? Averaged over leaf domains, 50.9% of domain assignments made from high alignment score alone are misclassifications, compared to 6.0% of domain assignments after thresholds are used to screen hits.

### Function and classification through specific domain assignments: Glycyl radical enzymes

To illustrate the effect of our proposed method, we examine domain assignments from the glycyl radical enzymes (RNR_PFL hierarchy). Its subgroups have distinct and important functions, including ribonucleotide reductases (RNRs), which synthesize deoxyribonucleotides, and pyruvate-formate lysases (PFLs), a family of catabolic enzymes. The proposed method places the sequence [Entrez:CAA42118] into RNR class 1 and places [Entrez: AAZ61477] into RNR class-1-like domain. The functions of these proteins are inferred by their subclass. Other proteins receive generic assignments to this family. For example, PFL2 (cd01677) is the best match to [Entrez:ABX41552] and [Entrez:EDQ26237] with alignment scores that fall short of the PFL2 threshold. The first alignment includes a long insertion (gap), and the latter exhibits weak sequence similarity; in both of these scenarios the transfer of functional annotation may not be straightforward.

Domain assignments also help to make biological insights. RNRs fall into classes that use different mechanisms and/or cofactors. Class 1 is oxygen dependent and class 3 is used by strictly or facultative anaerobic organisms. RNR_1_like has a similar active site to class 1 and at the time of curation, no specific literature was available about this subclass. We observed that the strictly anaerobic organism *Chlorobium limicola *DSM 24 has RNR_3 proteins (e.g. [Entrez:ZP_00512827]) as well as an enzyme ([Entrez:ZP_00512727]) that matches RNR_1_like, suggesting that RNR_1_like, a subfamily lacking experimental characterization, may contain non-oxygen dependent versions of RNR_1.

## Discussion

While many sequences can be classified by sequence similarity, profiles of protein domain families make it possible to quickly classify more distant homologs [23] and can better handle multi-domain proteins. An important step in transferring annotations from known protein families is identifying the subclass that provides the best characterization for the protein. Here, we conducted the first focused analysis of domain assignments from CDD in order to assess existing methods for domain and domain subfamily assignment and identify ways to improve the quality of assignments. We find that best-scoring hits are sometimes too specific, causing a sequence to be mislabelled by a subfamily of the correct domain. We propose a subclass assignment procedure that enables concrete assignments, computed quickly using existing data, and demonstrate that this procedure largely avoids over-predictions or false positive assignments and is robust enough to deal with situations such as incomplete hierarchies in which not all subfamilies have been identified. We elected to not employ standard jack-knife or cross-validation testing for a sequence against its correct domain, as the task is to classify sequence fragments that are very similar to a subfamily, where the subfamily model is also constructed from very similar sequences.

Although the sequence and domain databases evolve rapidly, we expect our findings to provide an accurate snapshot for some time. A version of our proposed method has been incorporated into the current version of the CD-Search program and the pre-calculated annotation of proteins with domains in NCBI's Entrez system. Domain assignments to specific orthologous subfamilies or ancient subfamilies are distinguished from non-specific assignments to a domain superfamily. High-confidence annotation of functional sites is also provided following these results. We hope the improved ability to quickly and accurately classify proteins will be a valuable step toward simplifying protein sequence analysis and the computational annotation of genomes.

## Competing interests

The authors declare that they have no competing interests.

## Authors' contributions

JF carried out the experiments and drafted the manuscript. AMB conceived the study, participated in its design, and helped to draft the manuscript.

## Supplementary Material

Additional file 1 **Extended Results section.** Extended presentation of all analyses, including additional data and discussion. Click here for file
