# Supplementary material for: Protein subfamily assignment using the Conserved Domain Database
Source: BMC Res Notes. 2008 Nov 14;1:114. doi: 10.1186/1756-0500-1-114 (PMC2632666; doi:10.1186/1756-0500-1-114)
Supplement: Additional file 1 — Extended Results section. Extended presentation of all analyses, including additional data and discussion. [file 1756-0500-1-114-S1.pdf]

Additional file 1

Correct domains have highest alignment scores

To assess the current default method for domain assignment, we benchmark the frequency of high alignment scores corresponding to the correct domain within the reference dataset. Among the 85609 representative sequences in NCBI-curated domain hierarchies with pre-computed alignment data, 23918 sequences have only self hits, 438 have only hits to other domains ("other hits"), and 61253 have both self and other hits. For sequences with only self hits or only non-self hits, a correct assignment is made by default to 98.2% of the sequences. For sequences with both self hits and other hits, the self hit has the highest score for 95.1% of sequences. Overall, highest scores alone would correctly assign domain models to 96.0% of sequence fragments in the test set. Additionally, for 91.5% of the domain models, high scores alone are sufficient to correctly classify every sequence member that is part of the model. That the correct domain so frequently has the high score may be surprising as NCBI-curated domain hierarchies may represent very fine-grained classifications and CD-Search often outputs many hits with close scores. Relatively complete alignments may contribute to the effectiveness of the best-score heuristic for the sequences in our benchmark dataset. While the pre-computed hits database contains many short, fragmented alignments between the PSSMs and protein sequences, over 99% of the self-hit alignments include at least 50% of the respective profile length. Length is a pre-computed property of each domain that approximately corresponds to the median domain footprint size of representative sequences used in the model, considering sequence weights. These results help to validate the distribution of sequences to curated domain models and confirm that RPS-BLAST bitscore is consistent with the metrics used to assign sequences to models.

Common sources of error in domain assignments

To determine the most common types of domain hits that produce erroneous assignments by scoring higher than the correct domain, we identify all sequences and domains with self hits and non-self hits. The other hits are categorized by their hierarchical relationships to the correct domain: parent/ancestor or child/descendant domains, representing the most-closely related domains, domains in other branches of the hierarchy, and domains in other hierarchies. Table 1 enumerates the sequences with particular combinations of self hits and other hits and the number of multiple hits and incorrect assignments based on the category of other hits, in order to provide the error rate for each category of other hits. The number of domains containing these sequences is provided as well, to indicate the prevalence of each type of error. Discriminating between domains and their immediate subclasses has the highest error rate, pointing out that identifying the ideal level of specificity is a challenge in CDD domain assignment. Child/descendant domains are the only category of domains from the same hierarchy that regularly score higher than the correct domain, doing so for 21.8% of sequences with self hits and hits to child or descendant domains. The low incidence of this case may be attributed to the relatively small number of internal domains. Higher scores from child/descendant domains may reflect a computational bias from longer profiles due to more sizable conserved regions, more homogeneous profiles, and other traits that lead to higher similarity scores. They may also reflect bias in CDD curation, as curators have been instructed to be cautious in assigning sequence fragments into specific subfamilies. Sequence fragments are left in parent or intermediate parent models when not associated unambiguously with a particular subfamily or child via phylogenetic analysis. A significant fraction of these cases may actually reflect correct biological classifications. This outcome may also, to some extent, reflect missing subfamilies in CD hierarchies that have not been declared due to insufficient data or taxonomic coverage. This

analysis also quantifies the number of overlapping domain models from different hierarchies, which may exist due to the reasons described in the Methods. The small number of errors from other hierarchies may be attributed to these overlaps.

Score thresholds complement high scores to identify correct domains

Because of apparent variations among hit scores from different domains, we assess the effect of using domain-specific threshold scores to screen incorrect domain hits. We define the threshold for each domain to be the lowest self-hit score from all sequences assigned to that domain in our reference dataset. First, we examine the propensity of incorrect hits to exceed either the threshold for the correct domain, or the threshold for the domain involved in the hit (Table 2). The proposed thresholds separate self-hit scores for one domain from the scores of almost all other hits to the domain's representative sequences. Only 9.1% of non-self hits exceed the threshold score for the correct domain. In contrast, 25.9% of the non-self hits score above their own domain threshold. The majority of these hits come from parent/ancestor domains, as expected if alignment scores for more specific domains tend to be higher. The hierarchical breakdown of correct and incorrect hits (Table 1; Table 2) indicate that high scores and threshold scores complement one another: child/descendant domains are the most common type to score higher than a self hit, and they rarely surpass their own domain thresholds. The converse holds for parent/ancestor domains. When requiring both the highest score and the domain-specific score threshold criteria, 99.0% of all specific assignments for the test set are correct.

Misclassifications error rate

The effect of domain-specific threshold scores may be better understood by quantifying misclassifications. We define *misclassifications* to be either descendants of the correct domain or domains that lie in other branches of the correct hierarchy. An incorrect assignment to a

parent/ancestor model is not necessarily a misclassification. For simplification, we disregard the small number of sequences whose best-scoring hits are to domains in other hierarchies and domains without high-scoring self hits within the hierarchy. The misclassification rate for a domain is the fraction of misclassifications over sequences assigned to domains within the correct hierarchy. Averaged over domains in multi-domain hierarchies, the misclassification rate using high-scores only is 2.6%. When score thresholds are used to eliminate low-scoring best hits, the misclassification rate drops to 0.85%.

Threshold scores reduce error due to missing subfamilies

Using misclassifications, we simulate a cross-validation experiment to explore the effect of incomplete domain hierarchies. We have assumed that curated domain hierarchies contain a representative and properly organized subset of members of the domain family. However, at any point in time, a hierarchy may not represent all ancient subclasses it intends to model, as the available sequence databases only provide a terse snapshot of protein domain diversity. It is not unlikely to encounter members of a domain family that do not belong in any of the subclasses that have been modeled explicitly. If we pretend that an existing domain model were missing from a hierarchy, what fraction of its sequence intervals have best hits to other models in the hierarchy that are not ancestors of the correct model? We approximate the effect of missing branches of a hierarchy by considering, for each leaf domain, hits to its representative sequences by other domains. Averaged over domains, 50.9% of domain assignments made from high alignment score alone are misclassifications. In other words, when a domain model is missing, there is nearly equal chance of classifying the sequence by a parent or ancestor of the correct domain, the desired outcome, and assigning the sequence to another branch of the hierarchy in error. Incorporating score thresholds to eliminate all hits that fall short of the respective domain’s

threshold score reduces the misclassification rate to a much lower 6.0%. For the misclassifications experiments, hits to domains from other hierarchies may be disregarded due to their rarity. Fewer than 4% of domains have any best-scoring hits to domains outside the hierarchy, even in the missing subfamily case.

Predicting correct domains for full sequence database

To assess the predictive power of the proposed algorithm, a standard cross-validation test would help to gauge the robustness of the domain models and their ability to identify more distant members. However, we have defined membership in each domain family or subfamily narrowly based on curator-defined models, so it may be more informative to verify that coverage is sizable. We assess whether the proposed rule makes specific assignments for a large fraction of likely members of the domains among all proteins in the Entrez database. For each domain, we survey up to 500 sequences chosen randomly among its pre-computed hits, which may number up to the tens of thousands. To simplify this experiment and reduce the number of specious hit, exclude the sequences used in the domain models, retain only the highest-scoring alignment of the domain to each sampled sequence, and consider only hits whose footprints span at least 50% of the domain length. For this experiment, the threshold scores are determined from sequences in our reference dataset. The proposed rule labels 27.4% of retained hits, averaged over domains, as correct domains, with a median of 12.2%. Pre-computed hits include many weak alignments that are unlikely to qualify as serious candidates for a specific domain assignment, suggesting that these rates may be as expected. These rates are equivalent to an average 885 sequences or a median 183 sequences, for each domain, based on the total number of hits to each domain. We find that a large fraction of the highest-scoring domain hits to their respective sequence intervals also meet the threshold score for the domain, and vice versa (data not shown). For example, for

81.5% of domains, at least 90% of the hits with alignment score above the domain-specific threshold are also the high scoring hit to that sequence region. By simulating the proposed rule onto realistic target proteins, this survey provides evidence that a large number of credible specific domain assignments will be made.
